# Evaluation of acceptability and use of lockable storage devices for pesticides in Sri Lanka that might assist in prevention of self-poisoning

**DOI:** 10.1186/1471-2458-9-69

**Published:** 2009-02-24

**Authors:** Keith Hawton, Lakshmi Ratnayeke, Sue Simkin, Louise Harriss, Vanda Scott

**Affiliations:** 1 Centre for Suicide Research, University of Oxford Department of Psychiatry, Warneford Hospital, Headington, Oxford, OX3 7JX, UK; 2 Sri Lanka Sumithrayo Rural Programme, Sri Lanka Sumithrayo, No. 60/7, Horton Place, Colombo 7, Sri Lanka; 3 Le Baradé, 32330 Gondrin, France

## Abstract

**Background:**

Self-poisoning with pesticides is a major reason for high suicide rates in rural areas of many developing countries. Safer storage of pesticides may be one means of prevention. We have conducted a study to assess the acceptability and use of lockable boxes for storing pesticides in rural Sri Lanka.

**Methods:**

Four hundred lockable metal storage boxes were given to farming households, 100 in each of four villages. Assessment interviews were conducted by Sumithrayo (NGO) field workers immediately after boxes were supplied (T1), 11 – 14 weeks later (T2), 30 weeks later (T3), and 18 months later (T4). Data on suicide and self-harm were collected from local police and hospitals.

**Results:**

At T1 only 1.8% (7/396) of households reported locking up pesticides, 72.5% (279/385) easy access to pesticides for adults and 50.4% (195/387) easy access for children. At T3 most informants in households using pesticides reported using the box all (82.3%, 298/362) or most of the time (7.2%, 26/362). Informants usually reported always locking the box (92.8%, 336/362) and most boxes were locked on inspection (93.6%, 339/362). By T4 there was some reduction in reporting that the box was kept locked all of the time (75.2%, 267/355) and the box being locked on inspection (73.8%, 262/355). Easy child access to the key was reported in relatively few households (10.7% at T4), although interviewers judged that this was possible in rather more (20.6%). Most informants regarded the box as useful (100% at T3 and 99.4% at T4), with convenience for storage, security, avoiding wastage, and protection of children being major factors. A message on the box about how to deal with bad feelings and the importance of safer storage was well received. The locks had been broken or the key lost in a few households.

**Conclusion:**

Introduction of lockable boxes for storing pesticides to farming households in Sri Lanka appeared to be acceptable. Most households used the boxes responsibly, although there was some decline in the proper usage over time. A large-scale trial of lockable storage devices in farming households in rural areas as a means of prevention of suicide and accidental poisoning is now indicated.

## Background

Rates of suicide are relatively high in several developing countries in Asia [1,2]. This is often due to particularly high rates in rural areas, particularly those where there are large numbers of small farms. One important contributory factor appears to be ease of access to agrochemicals, especially pesticides. They are widely used for self-poisoning in rural areas and most are relatively lethal when ingested [3]. In addition, there are also many accidental poisonings with these substances. It has been estimated that in excess of 250,000 – 300,000 deaths per year worldwide are due to pesticide poisoning [4-6], most of which result from intentional self-poisoning. This accounts for a substantial proportion of the estimated approximate suicide death toll worldwide of nearly 900,000 individuals per year [5]. However, the official number of suicides involving pesticides may be a considerable underestimate, as many deaths involving intentional self-poisoning with these substances may be misclassified in death statistics [7,8]. In addition there are a very large number of non-fatal poisonings with pesticides [9,10].

In Sri Lanka, the suicide rate increased very substantially from 1960 until recently. This rise in rates appears to have been almost entirely accounted for by increased self-poisoning with pesticides [11]. While environmental stresses may have been a contributory factor, the growth in use of pesticides is likely to have been an important element. Part of the reason for this is that pesticides are often kept around the household not locked up and hence freely accessible to suicidal individuals. It is estimated that in rural Sri Lanka 71% of suicide deaths involve pesticides. Many of those who take their own lives using pesticides are young [12]. Therefore this cause of death accounts for a very substantial burden to countries in terms of years of life lost [13]. It is essential that some means of reducing this unnecessary loss of life be found.

A range of approaches to preventing suicide by self-poisoning with pesticides has been proposed [5,14,15] These include reducing the availability of pesticides, especially those which are most toxic [16,17], public health campaigns, producing safer products, adding emetics or antidotes to pesticides, improved management of pesticide poisoning (e.g. first aid kits in villages for immediate treatment of poisoning, faster transfer to hospital, and improved hospital management), and provision of help at the community level to address issues such as domestic violence and alcohol abuse which may provoke acts of self-harm [3,9]. Encouraging safe storage of pesticides is one particularly important approach, especially as many acts of self-poisoning with pesticides appear to involve little premeditation. For example, in a study of people admitted to hospital with serious but non-fatal acts of self-harm in China, 83% of whom had ingested pesticides, more than one-third said that they had first considered the act only 10 minutes or less before carrying it out [10]. Of 268 self-poisoning patients interviewed in hospital in Sri Lanka, most of whom had taken pesticides, more than half had ingested the poison less than 30 minutes after deciding to self-harm [18].

Safer storage of pesticides could be effective because difficulty of access may reduce the desire to use this method for self-harm. Also, as suicidal crises are often brief, delayed or difficult access to a method may mean that the wish to carry out an act diminishes [19]. Whilst there is the possibility of turning to other methods, evidence from elsewhere suggests that where a popular method for suicide becomes less available substitution of method may be limited [20,21]. We are aware of two previous studies in Sri Lanka which have evaluated initiatives to encourage safer storage of pesticides [22,23], one of which had produced promising results [22].

In December 2004 Sumithrayo (a Sri Lankan national non-governmental organisation (NGO) concerned with helping distressed people and reducing suicide) implemented an initial pilot programme in two rural regions in Sri Lanka, in Kurunegala District in the North Western Province and in Hambantota District in the Southern Province, in which 100 lockable boxes for storage of pesticides were distributed to selected farming families in remote villages (50 families in 2 villages in the North Western Province and 50 in 2 villages in the Southern Province). Following encouraging signs about the acceptability of the boxes, a full study was designed. The main aim was to assess the acceptability and use of secure storage boxes.

## Methods

### Study Villages

Two villages in North Western Province of Sri Lanka (Kadawelagedera (371 households) and Wadumunna (282 households) and two villages in Southern Province (Dutugemunupura/Jayagama (438 households) and Devramvehera (270 households) were chosen to receive secure storage boxes. These villages were selected on the basis that they were in rural areas with farming being the main occupation, they were known to have relatively high rates of suicide and deliberate self-harm (DSH: non-fatal intentional self-poisoning) involving pesticides, and were accessible to Sumithrayo, the NGO involved in the study, but had received no prior intervention by this organisation.

The villages were relatively poor, especially those in the Southern Province. In the North Western Province rice is generally grown between mid-March and the end of July, and again from the end of September until February. Rain in Southern Province between the end of September and February allows rice to be grown then, along with bananas and a few cash crops. If water reservoirs are full there may be another season of rice growing. In villages in both areas pesticides can be bought in the nearest small town or the village shop.

### Recruitment

The study began in March 2006. For the purposes of this study, the definition of 'pesticide' included any herbicide, insecticide, fungicide, rhodenticide, or nematicide. In each of the four villages, all farming households that used pesticides were identified by the office of the village headman. Each such household in a village was assigned a unique number. A list of 100 potential participating households in each village was created by the temple priest, village elders and headman, through drawing 100 numbered slips of paper from a container. An additional 10 numbers were drawn to form a reserve list. The first 100 households from each village were visited by a member of Sumithrayo staff, who explained what the project was about and invited the householders to take part. An information sheet providing further details of the project was given to each household. If a household declined to participate, Sumithrayo staff visited the next household from the reserve list. If a household agreed to participate, the head of the family was asked to sign a consent form. The secure storage boxes were given out to the participating families at the village headman's office or temple. The Sumithrayo staff gave instructions on positioning the boxes and discussed possible locations. Where someone in the household was known to have been suicidal Sumithrayo staff asked that someone else in the household held the key.

### The boxes

Each box was made of 24 gauge steel and had ventilation holes in the sides and rear. The dimensions of the box were 45 cm by 32 cm by 22 cm. This size of box was used following complaints by some farmers that the box used in the pilot study was too small and consideration that a larger box than this might be too heavy to be supported by mud walls. A dividing shelf in the box was omitted for the same reason. A simple message was displayed on the door, which translates as: '*Poison is not the answer for anger, pain and despair. Talk to a trusted friend about your feelings of anger and sadness. Remember to keep all pesticides and poisons out of reach, safely locked in this box*.' The box was supplied with a single padlock and key, and with nails to fix it to a wall. There were four wall mounting brackets.

### Data collection

Information on box usage and pesticide storage was collected through interviews with participating households. The interviewers were eight paid Sumithrayo field officers. They all received training in the interview procedure and ongoing supervision. Information on episodes of suicide and deliberate self-harm (self-poisoning or self-injury) in the experimental and control villages was collected through regular searching of local hospital records, and through contact with local police (for suicides).

### Box use and pesticide storage

Following provision of the secure storage boxes, Sumithrayo staff visited participating households to conduct four semi-structured interviews:

i) Time 1 interview – within first week of receiving the box

ii) Time 2 interview – 11–14 weeks after receiving the box

iii) Time 3 interview – approximately 30 weeks after receiving the box

iv) Time 4 interview – 18 months after receiving the box

### Development of interview schedules

The interview schedules were developed through an initial workshop held in Sri Lanka with experts in pesticide poisoning, representatives of the agrochemical industry, suicide researchers, Sumithrayo staff and the project coordinator. Use was also made of information collected through the initial pilot study and questionnaires that had been developed for an earlier similar study elsewhere in Sri Lanka [22].

The interview schedules were developed in English. They were then translated into Sinhalese and subsequently back-translated. Back-translations were checked by staff at the Centre for Suicide Research at Oxford University and changes made if the meaning of questions had altered from the original, followed by further translation.

All interviews included a mixture of closed and open-ended questions. At the Time 1 interview information was collected about individuals in the household, family history of poisoning, deliberate self-harm (DSH) or suicide, current farming practice, pesticide use, storage and disposal of empty containers prior to receiving the box, and attitude to receiving the box. Respondents were also asked about the ease with which adults and children could access the pesticides. At the Time 2–4 interviews information was collected regarding box use and location, ease of access of adults and children to the box, pesticide storage, attitudes to box, problems with using box and attitudes to the message on the box. In addition, at the Time 3 interview information was obtained about socio-economic status and education level and the amount respondents would have been willing to pay for box if this had been required. At the Time 4 interview questions were also asked regarding the current position of the box and preference for different pesticide storage methods. At this interview the interviewers made their own assessment of how easily children might access the pesticides, dependent partly on the ages of the children (i.e. their height), where the box was located, and whether the box was kept locked and the key hidden. Sumithrayo staff had no other significant contact with the households during the study period.

### Data on suicide or self harm

At the Time 1 interview respondents were asked about any episodes of self-poisoning or other self-harm by family members. Data on subsequent self-harm and suicide were collected from local police and hospitals and through inquiry at the Time 2 to Time 4 interviews.

### Data entry and analysis

Interview data and information on episodes of suicide and deliberate self-harm were entered into Microsoft Access databases. All verbatim responses to open-ended interview questions were translated into English. The data were transferred into SPSS for analysis. The data analysis consisted of descriptive statistics and comparisons between groups using χ^2^, plus qualitative analysis of verbatim responses in order to identify themes.

The protocol for this study was approved by the Colombo Medical Ethics Committee.

## Results

### Characteristics of the households receiving storage boxes

More than three-quarters (318/400; 79.5%) of households included four or more persons. A similar proportion included children aged 0 to 18 years (281/400; 70.3%). In 36 (9.0%) of all the households included in the study at Time 1 the interviewee reported that a member of the household had been admitted to hospital after pesticide poisoning prior to commencement of study, 15 being reported as intentional and 21 as accidental. Six households reported that a member had died from pesticide poisoning, all with deliberate intent.

The land area (including garden) cultivated by the households was as follows: under 2 acres – 132 households (33.0%), 2–3 acres – 172 (43.0%), over 3 acres – 96 (24.0%). At the start of the study the predominant crops grown by the households showed marked variation between the households in the North Western Province and those in Southern Province, with rice and betel leaves being grown far more often by the households in the North West and peanuts, green grams and sesame being grown more often by households in the South. Livestock were farmed by 143 (35.8%) households, 94 (47.0%) in villages in the North West and 49 (24.6%) of those in the South. The most frequently farmed livestock were: cattle (114 households), chickens (32), pigs (8), buffalo (6) and goats (5).

At the Time 1 interview in only 7 out of 396 (1.8%) households were pesticides reported to be locked up. Easy accessibility to pesticides for adults was reported in 279/385 (72.5%) households, and for children in 195/387 (50.4%) households.

Dissatisfaction with current method of storage of pesticides was reported in nearly all households at the Time 1 interview (362/400, 90.5%). Analysis of the verbatim reasons given for lack of satisfaction with current method of storage show that the most frequent were fear of accidents or danger to life (34.0%), general lack of security (33.1%), risk of wastage or damage to pesticides (21.0%), and danger of suicide or non-fatal self-poisoning (18.2%).

### Households included in evaluation of boxes

**Figure 1:**
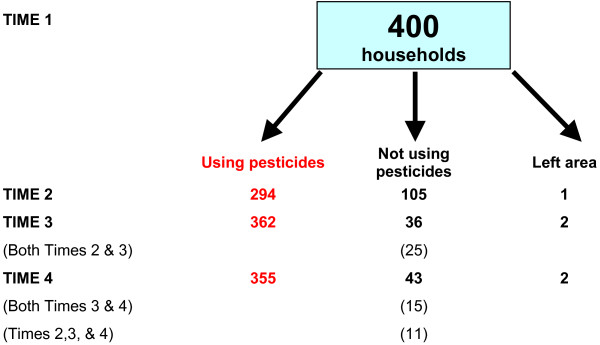
**Number of households in the study and pesticide use at the time of the interviews**.

Figure 1 shows the usage of pesticides in the households included in the study at the first and follow-up interviews. Far fewer households were using pesticides at Time 2 than at Time 3 because the season during which the Time 2 interview was conducted was a time of reduced agricultural activity. Because of this most of the results we present concern information collected at Time 3 and Time 4, and comparison with Time 1.

Often the farmer was not present in the household at the time of interview and another member of the household (usually their spouse) therefore participated. This applied more often at the Time 3 and Time 4 interviews. The farmer was unavailable for interview on all four occasions in 55 households.

In assessing the impact of the introduction of the storage boxes the analysis was restricted to the households which were using pesticides at the time of the interviews: i.e. Time 2 – 294; Time 3 – 362; Time 4 – 355.

### Location of the storage boxes

According to information collected at Time 3 the majority of households had sited the box outside the house: on a wall outside the house (253, 69.9%); separate building or shed (58, 16.0%). In a small proportion the box was kept inside the house (51, 14.1%). At Time 4 the box was in the same position in most households (317/355, 89.3%). Of the modified positions of the box, the most frequent was to inside the house (15/38, 39.5%). Building work was the usual reason for moving the box (N = 20), but in some cases the position of the box had been regarded as inconvenient (N = 7), and in others the box had fallen off a wall (N = 6).

### Impact of introduction of storage boxes

The introduction of the boxes had a major impact on the subsequent storage of pesticides, with most informants at the Time 3 and Time 4 interviews reporting that pesticides were being stored in the box, and few reporting storage in other locations (Table 1). However, at the Time 4 interview more reported storing pesticides in a field than at Time 3.

**Table 1:** Storage of pesticides before and after the introduction of the boxes^1^

	**Time 1** **(N = 400)**		**Time 3** **(N = 362)**		**Time 4** **(N = 355)**	
	**N**	**(%)**	**N**	**(%)**	**N**	**(%)**
**Box (locked away)**	(6)	(1.7%)	357	(98.6)	343	(96.6)
**Garden**	185	(51.5)	2	(0.6)	3	(0.8)
**Field**	140	(39.0)	6	(1.7)	25	(7.0)
**Separate building**	71	(19.8)	2	(0.6)	1	(0.3)
**House**	68	(19.0)	6	(1.7)	6	(1.7)
**On top of/under box**					4	(1.1)
**Other**	3	(0.9)	2	(0.6)	2	(0.6)

^1 ^Multiple responses could be given, hence the percentages add up to greater than 100%

At Times2, 3 and 4 informants in most households reported using the box all of the time (Time 2: 166/294, 56.5%; Time 3: 298/362, 82.3%; Time 4: 228/354, 64.4%) or most of the time (Time 2: 68/294, 23.1%; Time 3: 26/362, 7.2%; Time 4: 90/354, 25.4%). However, reported usage of the box in these and other households would have partly reflected the seasonality of pesticide use. At Time 4 only 7/354 (2.0%) of households reported that they never used the box. Reasons for never using the box included pesticides being hidden in the field (N = 3), fear of a family member attempting to gain access to the box and ingest the pesticides (N = 2), box fell down (N = 1) and not known (N = 1).

At the Time 2 and Time 3 interviews nearly all the informants reported that the box was always kept locked (Table 2). Inspection of the box at the time of the interviews showed that nearly all the boxes were locked. At the Time 4 interview, compared to Time 3, there was some reduction in the proportion of households reporting that the box was always kept locked (χ^2 ^= 26.13, df 3, p < 0.001), which was reflected in the reduced proportion locked on inspection (χ^2 ^= 52.04, p < 0.001) (Table 2). Reasons given at the Time 4 interview for not locking the box included: key lost or lock broken (N = 8), did not see the need to lock the box (N = 5), locking the box was a nuisance (N = 1), box was damaged (N = 1), and box not being used (N = 1) (not known – N = 3).

**Table 2:** Extent to which box locked according to informants and on inspection (Times 3 and 4)

**Box locked**	**Time 2** **(N = 294)**		**Time 3** **(N = 362)**		**Time 4** **(N = 355)**	
	**N**	**(%)**	**N**	**(%)**	**N**	**(%)**
**Informants:**						
**All of the time**	270	(91.8)	336	(92.8)	267	(75.2)
**Most of the time**	16	(5.4)	10	(2.8)	32	(14.6)
**Occasionally**	3	(1.0)	8	(2.2)	17	(4.8)
**Never**	5	(1.7)	8	(2.2)	19	(5.4)
**On inspection**	276	(93.9)	339	(93.6)	262	(73.8)

### Access to the box by children

Whereas at the initial interview half the informants (176/350, 50.3%) said that a child could easily get access to the pesticides, at the Time 3 interview only 9 (9/361, 2.5%) informants said that a child could easily get access to the key to the box (χ^2 ^= 210.87, p < 0.001). At Time 4 the proportion of households in which the informant said a child had easy access to the key had increased somewhat (37/346, 10.7%). The interviewer thought that a child could easily gain access to the key in considerably more households (64/311, 20.6%), although this assessment was not made for a substantial number of households.

### Attitudes to box

Nearly all the informants at the Time 4 interview said that they thought that the box was useful (353/355, 99.4%) and safe (353/355, 99.4%). Just 4 (1.1%) said that the box was inconvenient.

Content analysis of the informants' comments on the advantages of the box at Time 3 and Time 4 indicated that the most frequent were the convenience for keeping pesticides, especially having them all in one place, general security of the pesticides, and avoiding wastage or damage to the pesticides (Table 3). At Time 4 somewhat fewer respondents commented on the convenience for storing pesticides, but more of the comments were about avoiding wastage/damage to pesticides, protection for children, protection against theft, avoiding easy or hurried access and saving money. While relatively few specifically highlighted protection against suicide or DSH, other responses such as avoiding easy or hurried access may have implied the same thing.

**Table 3:** Advantages of the box (based on comments at Time 3 and Time 4)^1^

	**Time 3 (N = 361)**		**Time 4 (N = 355)**	
	**N**	**(%)**	**N**	**(%)**
**Convenience/all in one place**	178	(49.3)	123	(34.6)
**General security**	155	(42.9)	162	(45.6)
**Avoids wastage/damage to pesticides**	87	(24.1)	124	(34.9)
**Protection for children**	43	(11.9)	82	(23.1)
**Protection against accidents/protects lives**	32	(8.9)	39	(11.0)
**Protection against theft**	23	(6.4)	43	(12.1)
**Cannot be accessed easily/in a hurry**	11	(3.0)	40	(11.3)
**Feelings of safety/relief**	11	(3.0)	15	(4.2)
**Saves money**	8	(2.2)	26	(7.3)
**Protection against suicide/DSH**	6	(1.7)	12	(3.4)
**Saves time**	5	(1.4)	5	(1.4)
**Protection for animals**	2	(0.6)	6	(1.7)
**Other**	6	(1.7)	11	(3.1)

^1 ^Multiple responses could be given, hence the percentages add up to greater than 100%

There were far fewer comments about possible problems with the use of the box. The main one was that the box was not big enough to store all the pesticides that might be used by the household, with substantially more informants saying this at the Time 4 interview (Table 4). Five householders reported at the Time 4 interview that the padlock had broken. Four thought that having the pesticides all in one place might make them more readily accessible to thieves and suicidal people.

**Table 4:** Problems with use of the box (Time 3 and Time 4)

	**Time 3 (N = 361)**		**Time 4 (N = 355)**	
	**N**	**(%)**	**N**	**(%)**
**Not big enough**	22	(6.1)	54	(15.2)
**Padlock broken**			5	(1.4)
**Pesticides in one place may increase risk**			4	(1.1)
**Inconvenient location**	3	(0.8)	4	(1.1)
**Hard to hide key**	1	(0.3)	3	(0.8)
**Unable to find key**	1	(0.3)	3	(0.8)
**Keyholder unavailable**	1	(0.3)	1	(0.3)

When asked specifically at the Time 4 interview more than half the informants said that other villagers would like to have a box (209/344, 60.8%). However, only 5 (5/336, 1.5%) said that other villagers had made a box for themselves.

At the Time 4 interview five informants (5/355, 1.4%) reported attempted forced entry to the box. In two cases an individual who was apparently suicidal had tried unsuccessfully to force entry to the box (the informants commenting that the box may have saved the person's life), and in another case family members had to break into the box to get pesticides for urgent spraying because the farmer had gone out of the village and taken the key with him. Another case was reported by a family which was not using pesticides at the Time 4 interview – a household member had broken the box and swallowed pesticides, but recovered later in hospital.

Positive attitudes to the message on the box were indicated by most informants at the Time 3 (N = 352) and Time 4 (N = 338) interviews. Some examples were:

"Even when you are angry enough to drink poison seeing the message calms you down."

"Gives us a feeling that life is valuable."

"Very useful to see it constantly. Good to know that you can speak about your pain of mind to somebody."

"When you read it you know that there is poison in the box."

"It is useful. Visitors to the house too read it and gain by it."

"You gain more from the message than from the box."

At Time 4, 61 interviewees commented that the message had faded and was illegible. Also, at the Time 4 interview some respondents indicated that they would like the message or label to be changed in some way, including adding a visual representation of the message and using bigger letters.

When asked directly at the Time 3 interview nearly all the informants indicated that the boxes might have positive benefits in terms of prevention of both accidents (357/362, 98.6%) and suicide (344/362, 95%). Some examples of comments made by informants about possible benefits and negative effects in terms of prevention of accidents were:

**Positive effects **(98.6%)

"When the pesticides are not just left around there will be no room for accidents."

"Because the box is locked accidents are less."

"Accidents have reduced in the whole village. I am not afraid now of the safety of our children."

"Accidents are avoided because the pesticides no more get mixed with food."

"Because it's installed high, less chance of taking the poison accidentally."

"If the person holding the key is careful with it accidents can be avoided."

**Negative effects **(0.9%)

"Box is of no use"

"Can get the pesticides elsewhere"

"He feels the protection is for the pesticides and not the people"

Examples of possible positive and negatives effects of the boxes in terms of prevention of suicide were:

**Positive effects **(95.0%)

"Although we are surrounded by many problems we do not fear suicide as the box is locked."

"Because of the delay in getting the keys suicides are prevented."

"Because the box is locked I have no fear now. I attempted suicide before."

"Unable to take poisons in a state of anger."

"Because they are unable to open the box quickly their anger subsides."

**Negative/no effects **(3.3%)

"Person can take yellow oleander even if the pesticides are locked."

"If the man who has the key gets angry he can always access the poisons."

"If a person is bent on suicide they can always use a different method or even break the lock."

"If there is no key, taking the poisons to commit suicide will be easy because all the poisons are available in one place."

"If a person wants to commit suicide they can always purchase the poisons from the shop. So it is necessary to do awareness programs."

The negative comments indicated awareness of possible substitution of another method for suicide, and ways of getting access to the pesticides. However, most respondents thought the boxes would have a positive effect in terms of suicide prevention.

### Impact of the box on storage of leftovers

At both the Time 3 and Time 4 interviews nearly all of the informants reported that leftover pesticides were stored in the box (Time 3: 358/362, 98.9%; Time 4: 342/354 (96.6%)). However, at Time 4 informants in 19 (5.4%) households reported either not using the box for the storage of leftovers, or keeping some additional leftovers elsewhere, for example on top of the box, or, if needed soon, in the field.

### Disposal of empty pesticide bottles

While there was little evidence from content analysis of responses at either the Time 3 or Time 4 interviews that the presence of the box substantially altered the ways in which households disposed of empty pesticide bottles, more interviewees reported storing the empty containers in the box or washing them (Table 5).

**Table 5:** Disposal of empty pesticide bottles (based on description)

	**Time 1** **(N = 356)**		**Time 3** **(N = 358)**		**Time 4** **(N = 355)**	
	**N**	**(%)**	**N**	**(%)**	**N**	**(%)**
**Throw aside**						
**Field**	91	(25.6)	75	(20.9)	76	(21.4)
**Garden/around house**	29	(8.1)	10	(2.8)	2	(0.6)
**Jungle**	19	(5.3)	11	(3.1)	20	(5.6)
**Unspecified location**	43	(12.1)	20	(5.6)	25	(7.0)
**Collect/pile up**						
**Garden/around house**	50	(14.0)	63	(17.6)	42	(11.8)
**Field**	36	(10.1)	54	(15.1)	39	(11.0)
**Unspecified location**	69	(19.4)	68	(19.0)	42	(11.8)
**Sell**	40	(11.2)	33	(9.2)	52	(14.6)
**Bury**	21	(5.9)	26	(7.3)	6	(1.7)
**Burn**	17	(4.8)	21	(5.9)	10	(2.8)
**Open pit/hole**	13	(3.7)	20	(5.6)	45	(12.7)
**Destroy**	13	(3.7)	5	(1.4)	7	(2.0)
**Wash**	9	(2.5)	10	(2.8)	39	(11.0)
**Reuse for another purpose**	7	(2.0)	23	(6.4)	13	(3.7)
**Keep in box**	1	(0.3)	11	(3.1)	24	(6.8)

### Willingness to pay for the boxes

**Figure 2:**
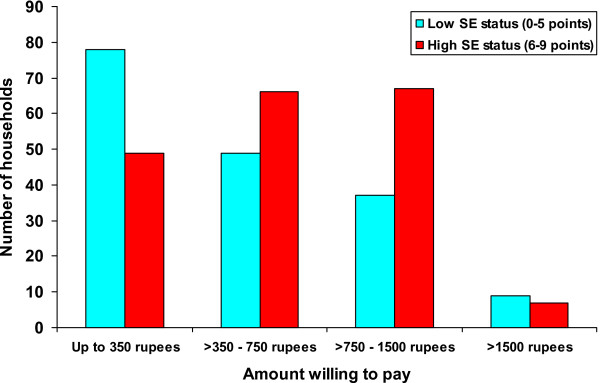
**Amount households were willing to pay for a box, according to socio-economic status**.

When asked at the Time 3 interview how much households would be willing to pay for the box if they needed to purchase it, there was wide variation in the amounts stated: Up to 350 rupees (N = 127, 35.1%); > 350 to 750 rupees (N = 115, 31.8%); > 750 to 1500 rupees (N = 104, 28.7%; > 1500 rupees (max 5000) (N = 16, 4.4%). As might be expected, the amount people were willing to pay co-varied according to socio-economic status (χ^2 ^= 17.37, 3 df, p = 0.001) (Figure 2). The socio-economic status of households was based on the number of the following items which were scored positive for each household: TV, refrigerator, tractor, water pump, complete house construction, brick or plaster walls, tiled or asbestos roof, land, and cultivated at least 2.5 acres (from Konradsen et al., 2007).

It should be noted that the amount most households were willing to pay for a box was less than the actual cost of producing each box (1832.5 rupees). Only 16 households were prepared to pay over 1,500 rupees for a box.

### Suicide and non-fatal deliberate self-harm

There were no suicides involving pesticides in the households with boxes after the boxes were introduced. One suicide involving another method occurred; this was poisoning with a chemical used by the individual in his occupation as a jeweller.

There were four episodes of DSH involving pesticides (one of these also involved consumption of yellow oleander seeds), plus 6 not involving pesticides (2 yellow oleander and 4 non-pesticide poisoning). In one of the episodes involving pesticides the storage box was forced open and the person managed to swallow a small amount of insecticide before the family could prevent him. In another case the individual consumed pesticides from another house. The source of the pesticide was not known for the other two cases.

## Discussion

Prevention of suicide and accidental deaths by pesticide poisoning requires multiple approaches [5,14,15]. Safer storage is one very practical potential measure. We have found that provision of lockable boxes to farming households appears to be a largely acceptable approach. Thus, while at the beginning of the study pesticides were kept locked up in very few households, 30 weeks after receiving the boxes informants in nearly all the households using pesticides reported that the pesticides were being stored in the box. The box was reported to be locked most of the time, which appeared to be confirmed by inspection of the box by the research interviewer.

There was, however, some indication of less consistent secure use of the box 18 months after their introduction. Thus somewhat fewer households reported using the box all the time and that it was always kept locked. Also on inspection by the interviewer the box was more often unlocked at the Time 4 interview (26.2%) than at the Time 3 interview (6.4%). Reasons for the box not being locked included the key being lost or the lock broken in a few cases and perceived lack of necessity to lock the box in some others. Pesticides were stored elsewhere (e.g. field) in a small number of households. There was also a small decrease by 18 months in the number of households storing leftover pesticides in the boxes. These signs of less consistent use of the box over time, which have also been found in a similar study in Sri Lanka [24], are important. They indicate that consideration must be paid to ensure more sustained secure storage over time. This may require community educational initiatives.

Most informants said the box was useful as a means of safer storage of pesticides. The most frequent reasons for this were convenience, especially having all the pesticides in one place, general security, and avoidance of wastage and damage to the pesticides. Importantly, since pesticides are often bought shortly before use, in most households it was reported that leftovers were being stored in the box, although as noted above there was a small decline in the number in which this was being done 18 months after the boxes were introduced. Ease of children gaining access to pesticides was also reported as reduced following the introduction of the boxes, although again there was some decrease in the strength of this finding 18 months after introduction of the boxes. Also, in the interviewers' opinions children could gain easy access to the key in more households than reported by the interviewees, although in approximately 80% of households easy child access was not thought to be an issue.

Unsurprisingly, the boxes had little apparent impact on disposal of empty pesticide containers. This is important since disposal is often haphazard and seemingly empty containers are likely to contain some pesticide residue, which might be a particular danger to children or other household members if bottles are used for some other purpose. However, there was some indication at the Time 4 interview that more households were storing empty containers in the box than previously.

Favourable responses were often reported regarding the message on the boxes, which encouraged help-seeking when people were distressed and safe storage of pesticides. However, it is not possible to say whether this message prevented acts of self-harm. If a similar message were to be used in a future project it would need to be more durable, since by the time of the 18 month interview some interviewees reported that the sticker had worn out or been damaged. Other changes, such as bigger letters and additional visual representation of the message might also be considered.

This study provides no direct evidence regarding the possible role of the boxes in the prevention of suicide or non-fatal poisoning, the numbers of households and hence the number of self-harm episodes being far too small to assess such an impact. A full-scale evaluation of this strategy would require careful evaluation of possible substitution of methods used for suicidal behaviour, particularly in view of ready access to yellow oleander, the seeds of which are highly toxic [25], in the areas where this study was conducted.

### Strengths and limitations of the study

The limited size of the study means that its main purpose was evaluation of acceptability and use of the storage boxes. We cannot conclude anything about the impact of such a device on suicide and self-harm. Nevertheless, the largely positive responses and indications of use suggest that this may provide a positive approach to the problem of poisoning with pesticides.

In assessing acceptability and use of the boxes we have relied mainly on informants' responses to questions. Social desirability may have influenced responses. Discrepancy in answers to questions about child access to the key to the padlock and more objective interviewer assessment of this indicates that the responses may have provided a somewhat exaggerated positive indication of outcome. The marked contrast in pattern of storage of pesticides after the introduction of the boxes compared to previously does nonetheless suggest a relatively substantial effect.

### Possible future developments in safer storage of pesticides

The current cost of the storage boxes is clearly prohibitive in terms of farmers themselves purchasing a box, especially those with low incomes. However, construction with plastic instead of metal would result in a far cheaper option. This would also allow production of a larger box if necessary, which was an issue for a substantial number of households (15.2%) by the time of the 18 month interview. Plastic boxes would also be lighter and therefore present less risk of damage to mud walls of houses if suspended, with less risk of them falling off. Wooden boxes are probably less desirable because of risk of damage by termites [24].

Our results are similar to those of Konradsen and colleagues [22], who assessed the introduction of either metal or wooden boxes to 200 farming households in two villages in North Central Province of Sri Lanka. These authors expressed concern, however, that encouraging farmers to store pesticides at home rather than in fields, as happened in a substantial proportion of households in both studies, might increase the risk of impulsive self-poisoning with pesticides. While we did not find evidence to support this, development of a lockable storage device that could either be fixed to a wall or partially buried (in a garden or field) would probably be advantageous. Farmers generally seem keen to keep secure pesticide containers in or near the house for reasons of security [24]. However, to avoid this possibly increasing risk of self-poisoning, maintenance of the security of the storage box is essential (see below). Since keys to locks in some households in both studies were lost and in others locks were broken, use of a combination lock may be advantageous [24], and would be likely to reduce potential child access to the contents of the box.

One important question is the extent to which induction of households regarding use of the boxes and possible further support might encourage effective box utilisation by households. In another study [23] in which 500 wooden storage boxes were distributed to farmers in Sri Lanka the box use was considerably less than in either our study or that of Konradsen and colleagues [22]. This outcome was attributed partly to lack of community involvement or support. In both our study and that of Konradsen and colleagues the communities were highly supportive of the projects and early interviews after provision of the boxes may have encouraged use of the boxes. It is also possible that subsequent research interviews may have assisted this process. While extensive support will not be feasible in any large-scale implementation of secure storage of pesticides, careful attention given to induction of communities and households regarding the importance and maintained use of storage devices is likely to be crucial.

## Conclusion

We believe the results of this study, together with those of Konradsen and colleagues [22], justify a large-scale trial of lockable storage devices in rural farming areas with high rates of suicide and accidental and non-fatal poisoning involving pesticides. This should be sufficiently powered to assess the impact on these outcomes, including any possible substitution of method for self-harm. Were such a trial to indicate a positive outcome this would support the large-scale introduction of this approach in rural areas of developing countries which have similar experiences with intentional pesticide poisoning. While it represents but one approach to prevention of the loss of life from pesticide poisoning, it could make a significant contribution to this major problem.

## Competing interests

KH, LR and VS received funding from Syngenta to attend meetings related to the project and Syngenta gave financial support to the project.

## Authors' contributions

KH and LR had the idea for the project and they, LH, SS and VS participated in designing it. LR (and her colleagues in Sumithrayo) collected the data, with support from KH and VS. The data were analysed by SS and LH. KH drafted the report and all authors contributed to the revisions and read and approved the final version.

## Pre-publication history

The pre-publication history for this paper can be accessed here:


